# Synthesis of New Fused Benzothiadiazepines and Macrocyclic Sulfamides Starting from *N,N*-Disubstituted Sulfamides and *N(Boc)*-Sulfamides

**DOI:** 10.5402/2012/810938

**Published:** 2012-05-16

**Authors:** Mohamed Dehamchia, Zine Regainia

**Affiliations:** ^1^Chemistry of Heterocycles Group, Laboratory of Applied Organic Chemistry, Department of Chemistry, Faculty of Sciences, University of Annaba, P.O. Box 12, Annaba 23000, Algeria; ^2^Department of Chemistry, Katholieke Universiteit Leuven, Celestijnenlaan 200F, 3001 Leuven, Belgium

## Abstract

Herein, we describe an efficient one-step synthesis of new fused benzothiadiazepine-1,1-dioxides and macrocyclic sulfamides. The synthesis of these compounds was achieved in moderate yields starting from previously described *N,N′*-disubstituted symmetric sulfamides and *N*-*tert*-butoxycarbonyl, *N′*-alkyl sulfamide. The chemical structures of all the new compounds reported in this work were confirmed by NMR, IR, and mass spectrometry. These compounds are beneficial building blocks that can be used in deriving new chemical entities that exert a wide spectrum of pharmacological activities.

## 1. Introduction

Cyclic sulfamides and their analogues have been the subject of many organic and medicinal chemistry studies due to their interesting biological activities that include anti-HIV and serine protease [1–4]. In addition, some cyclic sulfamide derivatives have been reported as nonhydrolyzable peptidomimetics [5, 6], metalloprotease inhibitors [7], and constrained peptides [8–10].

**Figure 1:**
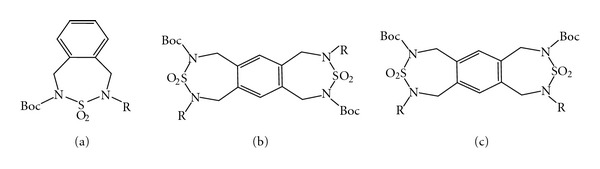
Fused benzothiadiazepine derivatives.

The benzothiadiazepine ring system has been considered as cyclic sulfamides, and these derivatives have been the subject, especially in the field of medicinal chemistry, because many useful therapeutic agents contain this heterocyclic system. For example, the nevirapine analogs, the pyrrolo[1,2-b][1,2,5]benzothiadiazepine-1,1-dioxides (PBTDs) and the pyrrolo[2,1-*d*][1,2,5]benzothiadiazepine-1,1-dioxides, were tested and reported as potential nonnucleosidic reverse transcriptase inhibitors [11]. Furthermore, the pyrrolo[1,2-*b*][1,2,5]benzothiadiazepine (PBTD) derivatives were also reported to exert potent anticancer activities [12]. Considering the diverse biological properties of this class of compounds and as part of continuous work on the synthesis of biologically active heterocycles [13], we herein report simple and efficient procedures for the synthesis of a new class of fused benzothiadiazepine derivatives (a), (b), (c) (Figure 1). These derivatives include two thiadiazepine rings and macrocyclic molecules containing a sulfamide functionality (–N–SO_2_–N–), which were synthesised using previously described *N*,*N* ^**'**^-disubstituted symmetric sulfamides and *N*(Boc), *N*′(alkyl)sulfamide [14–18].

In particular, we report the synthesis and spectroscopic properties of novel macrocyclic rings containing the sulfamide unit, which was incorporated by a direct reaction between *m*-dibromomethylbenzene derivatives and *N,N′*-disubstituted symmetric sulfamide. This strategy provides a ready access to a broad range of products. Beyond their pivotal role in the development of supramolecular chemistry [19, 20], this class of molecules has also served as the basis for designing various receptors of organic molecules [21]. Moreover, they have become useful building blocks for constructing nanoporous structures [22, 23].

## 2. Results and Discussion

**Scheme 1:**
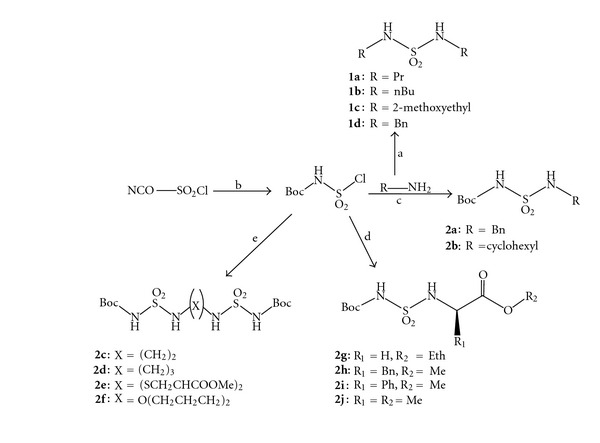
General synthesis of *bis* sulfamides and N(Boc)-sulfamides.* Reagents and conditions*: (a) SO_2_Cl_2_ (1/4 equiv), CH_2_Cl_2_, 24 h (b) *tert*-BuOH, CH_2_Cl_2_, 0°C (c) Amine, TEA, CH_2_Cl_2_, 0°C to rt (d) *α*-amino ester, TEA (2 equiv), CH_2_Cl_2_, 0°C to rt (e) diamine (0.5 equiv), TEA (1 equiv), CH_2_Cl_2_, 0°C to rt.

Our earlier studies involved the synthesis of heterocyclic compounds containing sulfonyl groups [9, 10, 15, 16]. Chlorosulfonyl isocyanate (CSI) and sulfuryl chloride (SO_2_Cl_2_) have been shown to be versatile reagents in the synthesis of heterocyclic chemistry. They have been used in the direct introduction of sulfonyl groups into heterocycles. Several total syntheses of *N*,*N*′-disubstituted symmetric sulfamides (**1a-d**) have been reported in the literature including the original synthetic approaches [24–27]. Thus, the starting material, sulfuryl chloride, was treated with an excess of the corresponding amine in dichloromethane for 24 h (Scheme 1), and this resulted in the formation of products **1a-d** in moderate yields. The synthesis of the key intermediates *N(Boc)*, *N*′-alkyl-sulfamide (**2a-f**) and* N-*((Boc)sulfamoylamino)carboxylates (**2g-j**) was accomplished as shown in Scheme 1. The carbamylation of chlorosulfonyl isocyanate with *tert*-butyl alcohol at 0°C in dichloromethane followed by *in situ* sulfamoylation with the corresponding amine, amino acid ester hydrochloride, or diamine in the presence of triethylamine (TEA) gave the desired *N(Boc)*, *N*′(alkyl)sulfamide (**2a-b**), *N*(Boc), *N*′-sufamoylamino acid esters (**2g-j**) or *bis*-carboxylsulfamides (**2c-f**) [28, 29].

**Scheme 2:**
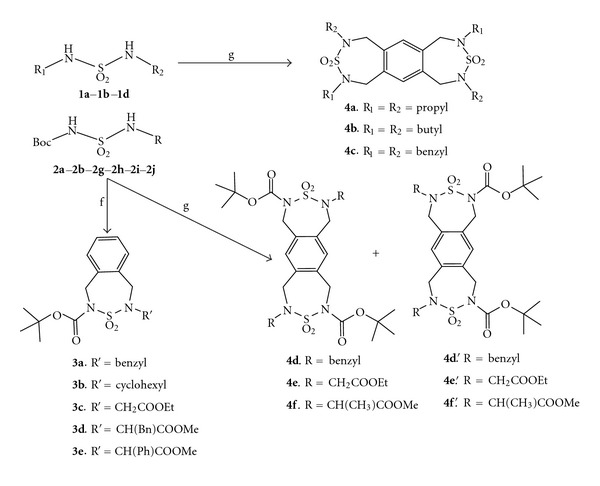
General synthesis of tricyclic benzothiadiazepine-1,1-dioxides.* Reagents and conditions:* (f) *α*,*α*′-dibromo-*o*-xylene, K_2_CO_3_, CH_3_CN, reflux, 4 h (g) K_2_CO_3_, 2,3,4,5-tetrakis(bromomethyl)benzene, CH_3_CN, reflux 10 h.

As outlined in Scheme 2, the *N*,*N*′-disubstituted symmetric sulfamides (**1a-d**) are a suitable starting material for the synthesis of an array of new benzocondensed scaffolds (**4a-c**) in good yields 75–79%. The starting materials, *N*,*N*′-disubstituted symmetric sulfamides **1a-d**, were condensed with 1,2,4,5-tetrakis(bromomethyl)benzene (0.5 equiv) by refluxing in acetonitrile for 10 h in the presence of potassium carbonate (K_2_CO_3_) to afford fused Benzo-di-thiadiazepines **4a-c**.

In the second route, after replacing *N*,*N*′-disubstituted sulfamides by *N*(Boc)sulfamides derivatives (**2a-j**) under the same conditions, products **4d-f **and **4d′-f′** were formed. Both isomers (symmetric and asymmetric) were separated by flash chromatography using dichloromethane as an eluant. The products were obtained in different yields as summarized in Table 1. Notably, the percentage yields of the asymmetric fused benzothiadiazepines **4d′-f′** were relatively low. These yields seem to be strongly dependent upon the reaction conditions (solvent, temperature, and steric effect). Therefore, further optimization of the reaction conditions might improve the yield of these reactions.

The prepared *N*(Boc)-protected compounds (**2a-j**) have traditionally been a starting point for the design of novel benzocondensed derivatives (**3a-e**) by condensation with *α*,*α*′-dibromo-*o*-xylene in acetonitrile in the presence of potassium carbonate (K_2_CO_3_) (Scheme 2).

The presence of the *tert*-butoxycarbonyl (Boc) group, which activates the sulfamide nitrogen nucleophilicity, was required for substitution. This protecting group was removed by trifluoroacetic acid to yield the unprotected fused cyclic sulfamides [30]. These deprotected compounds were considered excellent starting materials for preparation of biomolecule analogues employing different types of reactions such as regioselective Mitsunobu reaction (DEAD, PPh_3_, THF at room temperature, 2 h) [31, 32]. The structures of fused compounds were confirmed by IR, mass spectrometry, and NMR (^1^H, ^13^C), and the results are presented in Table 1.

The IR spectra of compounds **3a-e** displayed the characteristic absorption bonds near 1370 for SO_2_, near 1140 cm^−1^ for SO_2_ and strong absorption in the vicinity of 1740 cm^−1^ due to C=O stretching. If the substituent R is an ester group, there must also be an intense stretch in the carbonyl region of the spectrum near to 1750 cm^−1^. At ambient temperature, the ^1^H-NMR spectra of the benzothiadiazepines showed sharp signals near 1.40 ppm indicating the presence of *Boc* group. The aromatic proton signals appear at 7 ppm as one multiplet of 4H for (**3a-c**) and 9H for (**3d-e**). ESI-MS spectra of the compounds **3a-e** showed ion peaks due to [M+Na]^+^ and [M+2Na]^+^.

The structures of fused **a**, **b,** and **c** compounds were supported by analysis of the mass spectra ESI-MS, which showed peaks respectively at *m/z* 509, 567, and 710 indicating molecular masses of ions [M+Na]**^+^**. As shown in Table 1, all the H^1^NMR spectra showed one singlet peak near 7 ppm, which is a strong indication of the presence of aromatic protons. In the infrared data, all spectra showed bands near 1150 and 1350 cm^−1^ due to SO_2_ stretching. In the ESI-MS spectra, all the prepared fused compounds **4d-f′** exhibit intense peaks corresponding to the molecular weight [M+Na]^+^. Since the symmetric and asymmetric fused compounds have the same molecular weight, it was difficult to extract all the rich structural information from the mass spectra. In the IR spectra (Table 1), there are peaks at about (1140–1170) and (1368–1390) cm^−1^, due to the sulfonyl group (SO_2_) stretching, and at about 1715–1732 cm^−1^, due to C=O stretching vibrations. For the compounds containing an ester group, IR spectra showed also bands near 1700 cm^−1^ due to C=O stretching. The one difference in the ^1^H NMR spectra between the symmetric and asymmetric fused is the aromatic region. The ^1^H NMR spectra of symmetric fused **4d-f** showed resonances attributed a two aromatic protons, which appeared as one singlet with a relative integration of 2 indicating the equivalency of the two hydrogens. However, for the asymmetric fused derivatives, the ^1^H-NMR spectra showed two different kinds of aromatic protons with relative integration of 1 : 1.

## 3. Synthesis of New Macrocyclic Sulfamides

**Scheme 3:**
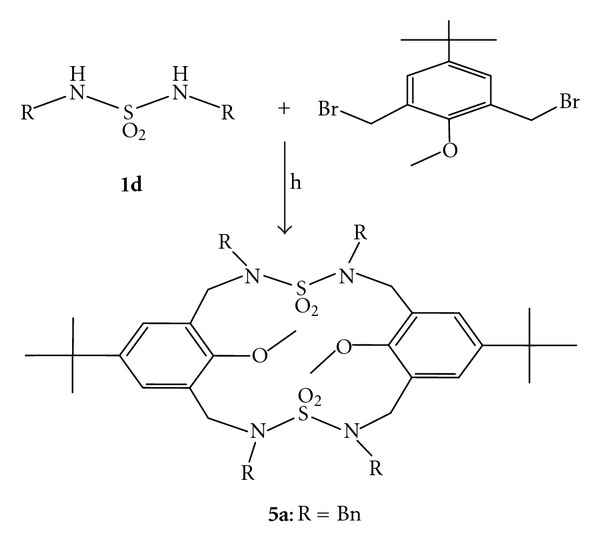
Synthetic pathway for the synthesis of macrocyclic sulfamide.* Reagents and conditions:* (h) K_2_CO_3_, CH_3_CN high-dilution, reflux 24 h.

There are many strategies available for the synthesis of benzylic amide macrocycles that involve the reaction of an ester group with an amino group [33–35]. In this work, we also investigated the synthesis of new macrocyclic containing the sulfamide unit. As shown in Scheme 3, the desired macrocyclic sulfamides **5** were synthesized in one step [2+2] condensation under high dilution conditions [36]. A solution of 1-methoxy-4-tert-butyl-(2,6-dibromomethyl)benzene (1 equiv) in 20 mL of acetonitrile and a solution of *N*,*N*′-disubstituted symmetric sulfamide (1 equiv) in 20 mL of CH_3_CN were added dropwise using two mechanically driven syringes over 5 h into solution of K_2_CO_3_ (4.5 equiv) in 130 mL of CH_3_CN under nitrogen with stirring at reflux for 24 h. The reaction mixture was subsequently cooled down, and the solvent was removed. Dichloromethane was added to the obtained crude, and this solution was washed with 2 N HCl then with water and dried with magnesium sulfate. The solvent was evaporated to give the macrocycle **5a** in 58% yield. In the macrocyclization reactions, it was critical to find suitable reaction conditions that maintain the correct condensation, while keeping the reactions fast enough to prevent buildup of reactive intermediates.

The structure of macrocyclic compounds **5a** was unambiguously confirmed by IR, mass spectrometry, and NMR (^1^H,^13^C) spectroscopy. The infrared spectrum showed characteristic bands at 1148 and 1361 cm^−1^, which were assigned to the sulfonyl group (SO_2_). A molecular peak of 952 [M+Na]^+^ was observed by ESI mass spectrometry. In addition, the 300 MHz ^1^H spectrum, measured on a sample dissolved in CDCl_3_, showed a relative integration of 4 : 20 for the two sets of peaks at 6.78 and 7.30–7.45 ppm. These signals were assigned to the aromatic regions of product.

## 4. Conclusion

In summary, we have successfully synthesized and characterized a new series of *N*-protected fused benzothiadiazepines, which offer good starting materials for the synthesis of new molecules with interesting biological activities. In the second part, we described an efficient method for the synthesis of new macrocycle with sulfamide moiety with potential diverse applications in supramolecular chemistry and as starting materials for further synthetic transformations. The synthetic example presented in this work is one of the simplest and most efficient macrocyclization reactions based on the technique of high-dilution conditions. The biological evaluation of the compounds synthesized in this work is currently being carried out.

## 5. Experimental Section

### 5.1. Instrumentals and Characterization

 NMR spectra were acquired on commercial instruments (Bruker Avance 300 MHz or Bruker AMX 400 MHz) and chemical shifts (*δ*) are reported in parts per million downfield from internal Me_4_Si (s = singlet, d = doublet, dd = double of doublet, t = triplet, q = quartet, m = multiplet). Mass spectrometry data were obtained with an HP MS apparatus 5989A, at 70 eV for EI spectra and with methane as reagent gas for CI spectra. The ESI-MS were obtained on Mariner (ESI TOF) and API 365 (ESI 3Q) mass spectrometers with methanol as a spray solvent. UV-Vis spectra were taken on a Perkin*­*Elmer Lambda 20 spectrometer. Melting points (not corrected) were determined using a Reichert Thermovar or Electrothermal 9200 Apparatus. The microwave oven was a monomode discover MW reactor. All reactions were done in a 10 mL glass tube sealed with a Teflon stopper unless stated otherwise.

### 5.2. General Procedure A for the Synthesis of *N*,*N*′-Disubstituted Symmetric Sulfamides **(1a-d)**

These compounds were prepared as described in the literature [15]. The reaction was carried out by dropwise addition of a solution of sulfuryl chloride (1 equiv) in 20 mL of dichloromethane to a solution of the corresponding amine (4–6 equiv) in 50 mL of CH_2_CL_2_ at 0°C in darkness. Gas evolution was observed during the addition. The reaction mixture was warmed to room temperature (rt), stirred for 24 h, and monitored by TLC (SiO_2_). The crude was washed by HCl (2 N, 2 × 20 mL) water (2 × 30 mL) and dried over Na_2_SO_4_. The solution was filtered and then concentrated under reduced pressure to leave yellow solid as the crude product. Column chromatography (CH_2_Cl_2_, MeOH 95 : 5) afforded the *N*,*N*′-dialkyl sulfamide.

***N*,*N*′-Dipropylsulfamide (1a)**

This compound was prepared according to the general procedure A, using a solution of propylamine (6 equiv) in CH_2_Cl_2_ and SO_2_Cl_2_ (1 equiv) in CH_2_Cl_2_. Yield = 60% (was obtained as a white solid); R*_f_* = 0.45 [SiO_2_, CH_2_Cl_2_/MeOH (95 : 5)]; Mp 64–65°C (described: 62–63°C). IR (KBr, *ν* cm^−1^): 3280 (NH), 1333 and 1150 (SO_2_). ^1^H NMR (CDCl_3_): 0.95 (t, J = 7.2 Hz, 6H, CH_3_), 1.57 (sext, J = J′ = 7.1 Hz, 4H, *β*-CH_2_), 2.99 (q, 4H, *α*-CH_2_), 4.27 (t broad, 2H, NH). ^13^C NMR (CDCl_3_): 11.26 (*γ*-C), 22.89 (*β*-C), 44.95 (*α*-C). LRMS (CI): 181 [M+H]^+^.

***N*,*N*′-Dibutylsulfamide (1b)**

Yield = 58% (was obtained as a white solid); R*_f_* = 0.36 [(SiO_2_, CH_2_Cl_2_))]; Mp: 126–127°C (described 126.5°C). IR (KBr, *ν* cm^−1^): 3281 (NH), 1314 and 1145 (SO_2_). ^1^H NMR (CDCl_3_): 4.33 (t broad, 2H, NH), 3.04 (m, 4H, *α*-CH_2_), 1.54 (m, 4H, *β*-CH_2_), 1.38 (m, 4H, *γ*-CH_2_), 0.93 (t, J = 7.1 Hz, 6H, CH_3_). ^13^C NMR (CDCl_3_): 43.2 (*α*-C), 31.7 (*β*-C), 20.11 (*γ*-C), 13.88 (CH_3_). LRMS (CI): 209 [M+H]^+^.

***N*,*N*′-Di*­*(2*­*Methoxyethyl)*­*Sulfamide (1c)**

Yield = 61% (was obtained as a viscous oil); R*_f_* = 0.36 [SiO_2_, CH_2_Cl_2_/MeOH (95 : 5)]; IR (KBr, *ν* cm^−1^): 3279 (NH), 1316 and 1147 (SO_2_). ^1^H NMR (CDCl_3_): 3.22 (q, 4H, *α*-CH_2_), 3.36 (s, 6H, CH_3_), 3.52 (m, 4H, *β*-CH_2_), 5.28 (t, 2H, NH), ^13^C NMR (CDCl_3_): 42.67 (*α*-C), 58.57 (CH_3_), 70.93 (*β*-C). LRMS (CI): 213 [M+H]^+^.

***N*,*N*′-Dibenzylsulfamide (1d)**

Yield = 59% (was obtained as a white solid); R*_f_* = 0.37 (SiO_2_, CH_2_Cl_2_); Mp: 182–184°C (described 180–182°C). IR (KBr, *ν* cm^−1^): 3270 (NH), 3034 (CH-Ar), 1350 and 1143 (SO_2_). ^1^H NMR (CDCl_3_): 4.17 (d, 4H, CH_2_), 4.37 (t broad, 2H, NH), 7.28–7.34 (m, 10H, Ar-H). LRMS (CI): 277 [M+H]^+^_,_ 199, 91.

### 5.3. General Procedure B for the Synthesis of *N*-tert-Butyloxycarbonyl, *N*′-Alkyl Sulfamide: Carbamoylation-Sulfamoylation **(2a-f)**

To a stirred solution of CSI (1 equiv, 10 mmol, 1.4153 g) in 20 mL of anhydrous dichloromethane at 0°C was added a solution of *tert*-butyl alcohol (1 equiv, 10 mmol, 0.7412 g) in 20 mL of dried CH_2_Cl_2_. After being stirred for 30 min, the resulting solution of *N(Boc)*-sulfamoyl chloride and triethylamine (TEA) in 20 mL dichloromethane was added dropwise to a solution of amine (1 equiv) or (diamine 0.5 equiv) in 20 mL of CH_2_Cl_2_. The reaction temperature did not rise above 5°C. The resulting reaction solution was allowed to warm up to rt over 3 h. The reaction mixture was diluted with dichloromethane and washed with 0.1 N HCl and brine. The organic layer was dried (Na_2_SO_4_) and concentrated *in vacuo* to give the crude product. Recrystallization from CH_2_Cl_2_ at low temperature afforded the expected compounds in 70–85% yield.

**2a** and **2b** were prepared according to the general procedure **B**; see [29].

***N*,*N*′-Bis(*tert*-Butoxycarbonylsulfamoyl)-1,2-Diaminoethane (2c)**

This compound was prepared according to the general procedure B using a solution of 1,2-ethan diamine (0.5 equiv, 5 mmol, 0.6010 g) in CH_2_Cl_2_. Yield = 70% (was obtained as a white solid); R*_f_* = 0.20 [SiO_2_, CH_2_Cl_2_-MeOH 95 : 5]; IR(KBr, *ν* cm^−1^): 3286 and 3311(NH); 1709 (C=O); 1346 and 1141 (SO_2_). ^1^H NMR (DMSO-d_6_): 10.90 (s, 2H, N**H**Boc); 7.65 (s, 2H, NH); 2.98 (s, 4H, CH_2_); 1.43 (s, 18H, *t*Bu). HRMS ESI^+^: *m/z*: 441 [M+Na]^+^.

***N*,*N*′-Bis(*tert*-Butoxycarbonylsulfamoyl)-1,3-Diaminopropane (2d)**

This compound was prepared according to the general procedure B using a solution of 1,3-propandiamine (0.5 equiv, 5 mmol, 0.3706 g) in dichloromethane Yield = 70% (was obtained as a white solid); R*_f_* = 0.25 [SiO_2_, CH_2_Cl_2_-MeOH 95 : 5]; Mp: 175–177°C. IR (KBr, *ν* cm^−1^): 3266 and 3212(NH); 1697 (C=O); 1348 and 1138 (SO_2_). ^1^H NMR (DMSO-d_6_): 10.79 (s, 2H, N**H**Boc); 7.52 (t, 2H, NH); 2.89 (q, 4H, CH_2_-N); 1.63 (m, 2H, CH_2_); 1.42 (s, 18H, *t*Bu). HRMS. ESI^+^: m/z: 455 [M+Na]^+^, 887 [M*2+Na]^+^.

**Compound (2e)**

This compound was prepared according to the general procedure B using a solution of L-cystine methyl ester dihydrochloride (0.5 equiv, 5 mmol, 1.7064 g) in CH_2_Cl_2_ and triethylamine (2 equiv, 20 mmol, 2.0238 g). Yield = 71% (was obtained as a white solid); R*_f_* = 0.37 [SiO_2_, CH_2_Cl_2_-MeOH 95 : 5]; IR (KBr, *ν* cm^−1^): 3289 and 3240(NH); 1709 and 1748 (C=O); 1364 and 1139 (SO_2_). ^1^H NMR (DMSO-d_6_): 10.98 (s, 2H, NHBoc); 8.40 (d, 2H, NH); 4.22 (q, 2H, CH); 3.66 (s, 6H, CH_3_); 3.00 (d, 4H, CH_2_); 1.41 (s, 18H, *t*Bu). HRMS ESI^+^: *m/z*: 659 [M+Na]^+^, 1275 [M*2+Na]^+^.

**Dimethyl-5,5′-oxybis(2-(*N*-(tert-butoxycarbonyl)sulfamoylamino)pentanoate) (2f)**

This compound was prepared according to the general procedure B using a solution of 1,3-propan diamine (0.5 equiv, 5 mmol, 0.661 g). Yield = 73% (was obtained as a white solid); R*_f_* = 0.49 [SiO_2_, CH_2_Cl_2_-MeOH 95 : 5]; Mp: 132–134°C. IR (KBr, *ν* cm^−1^): 3296 (NH); 1713 and 1738 (C=O); 1371 and 1143 (SO_2_). ^1^H NMR (CDCl_3_): 7.52 (s, 2H, NHBoc); 5.79 (t, 2H, NH); 3.57 (t, 4H, CH_2_–O); 3.20 (q, 4H, CH_2_–N); 1.84 (m, 4H, CH_2_); 1.49 (s, 18H, *t*Bu). HRMS ESI^+^: *m/z*: 514 [M+Na]^+^, 1004 [M*2+Na]^+^.

### 5.4. General Procedure C for the Preparation of *N*(Boc), *N*′-Sufamoyl amino Acid Esters **(2g-j)**

To a suspension of the amino acid ester hydrochloride (1 equiv, 10 mmol) was added triethylamine (1 equiv, 10 mmol, 1.0119 g) in 20 mL of dichloromethane. Simultaneously the *tert*-butyl chlorosulfonyl carbamate was prepared by addition of *tert*-butyl alcohol (1 equiv, 10 mmol, 0.7412 g) in 20 mL of CH_2_Cl_2_ to an ice-cooled solution containing CSI (1 equiv, 10 mmol, 1.4153 g) in 20 mL of dichloromethane. The obtained reagent solution was slowly added to the solution of amino acid ester hydrochloride in 30 mL of dichloromethane at the same time as of Et_3_N (1 equiv, 10 mmol, 1.0119 g). The reaction was monitored by TLC. The mixture was then diluted with CH_2_Cl_2_ (100 mL) and washed with 2 portions of 1 M HCl and water. The solution was then dried with Na_2_SO_4_ and concentrated *in vacuum* to give the crude product. Recrystallization from CH_2_Cl_2_ at low temperature or chromatography on silica gel (eluent: CH_2_Cl_2_/MeOH, 9 : 1) afforded the pure carboxyl sulfamide.

The synthesis of the compounds, starting from CSI, *tert*-butyl alcohol and methyl esters of amino acids (L-alanine** 2j** and L-phenylalanine 2 h) has been previously reported [9, 10].

**Ethyl [*N*,(*N*′-tert-Butyloxycarbonyl)-sulfamoyl]-Acetate (2g)**

This compound was prepared according to the general procedure C using a solution of glycine ethyl ester hydrochloride (1 equiv, 10 mmol, 1.396 g). Yield = 72% (was obtained as a white solid); R*_f_* = 0.60 [SiO_2_, CH_2_Cl_2_-MeOH 9 : 1]; Mp 122–123°C. IR (KBr, *ν* cm^−1^): 1352 and 1126 (SO_2_), 1735 and 1675 (C=O). ^1^H NMR (300 MHz, CDCl_3_): 1.5 (s, 9H, *t*Bu), 1.30 (t, 3H, CH_3_), 3.96 (d, 2H, N-CH_2_), 4.24 (q, 2H, CH_2_), 5.63 (t, 1H, NH-CH_2_), 7.25 (s, 1H, NHBoc). HRMS ESI^+^:* m/z*: 306[M+Na]^+^.

**Methyl [*N*,(*N*′-tert-Butyloxycarbonyl)-sulfamoyl]-2-phenyglycynate (2i)**

This compound was prepared according to the general procedure C using a solution of phenyl glycine methyl ester hydrochloride (1 equiv, 10 mmol, 2.0165 g). Yield = 76% (was obtained as a white solid); R*_f_* = 0.70 [SiO_2_, CH_2_Cl_2_-MeOH 9 : 1]; Mp 144–146°C. IR (KBr, *ν* cm^−1^): 1362 and 1141 (SO_2_), 1735–1712 (C=O). ^1^H NMR (300 MHz, CDCl_3_): 1.44 (s, 9H, *t*Bu), 3.74 (s, 3H, O-CH_3_), 5.27 (d, 1H, CH), 6.27 (d, 1H, NH), 7.36 (s, 5H, Ph), 7.44 (s, 1H, NHBoc). HRMS ESI^+^: m/z: 367 [M+Na]^+^.

### 5.5. General Procedure D for the Synthesis of Benzothiadiazepines **(3a-e)**

To a stirring solution of [*N*-*tert*-butyloxycarbonyl, *N*′-alkyl]-sulfamide (1 equiv, 1 mmol) in CH_3_CN (50 mL) in a 100 mL round bottom flask was added K_2_CO_3_ (2.5 equiv, 2.5 mmol, 0.34552 g) and *α*,*α*′-dibromo-*o*-xylene (1 equiv, 1 mmol, 0.26396 g). The resulting mixture was stirred at reflux for 4 hours then cooled to room temperature. The reaction mixture was filtered and the filtrate was evaporated under reduced pressure. The residue was dissolved in CH_2_Cl_2_, washed with 2 portions of HCl (1 M) and water and dried with Na_2_SO_4_, and the solvent was removed under reduced pressure to give the crude oil. Flash chromatography on silica gel CH_2_Cl_2_ to furnish the pure fused cyclic sulfamide in 70–85% yields the following.

**1,5-dihydro-2-tert-butoxycarbonyl-4-benzylbenzo[d][1,2,7]thiadiazepine-3,3-dioxides (3a)**

This compound was prepared according to the general procedure D, using a solution of** 2a **(1 equiv, 1 mmol, 0.28635 g). Yield = 71% (was obtained as a viscous oil); R*_f_* = 0.36 [SiO_2_, CH_2_Cl_2_]. IR (KBr, *ν* cm^−1^): 1389 and 1141 (SO_2_), 1732 (C=O). ^1^H NMR (300 MHz, CDCl_3_): 1.43 (s, 9H, *t*Bu), 4.14 (s, 2H, CH_2_-N), 4.37 (s, 2H, CH_2_-N-Boc), 4.91 (s, 2H, CH_2_-Ph), 7.03–7.37 (m, 9H, H-Ar). HRMS ESI^+^: *m/z*: 799 [M*2+Na]^+^.

**1,5-dihydro-2-tert-butoxycarbonyl-4-(cyclohexyl)benzo[d][1,2,7]thiadiazepine-3,3-dioxides (3b)**

This compound was prepared according to the general procedure D, using a solution of** 2b **(1 equiv, 1 mmol, 0.27837 g). Yield = 80% (was obtained as a white solid); R*_f_* = 0.42 [SiO_2_, CH_2_Cl_2_]; Mp 96–98°C. IR (KBr, *ν* cm^−1^): 1376 and 1145 (SO_2_), 1726 (C=O). ^1^H NMR (300 MHz, CDCl_3_): 1.23–1.72 (m, 10H, Cyclohexyl); 1.41 (s, 9H, *t*Bu), 3.94 (m, 1H, CH), 4.58 (s, 2H, CH_2_), 4.88 (s, 2H, CH_2_), 7.20–7.28 (m, 4H, H-Ar). HRMS ESI^+^: *m/z*: 784 [M*2+Na]^+^.

**1,5-dihydro-2-tert-butoxycarbonyl-4-((ethoxycarbonyl)methyl)benzo[d][1,2,7]thiadiazepine-3,3-dioxides (3c)**

This compound was prepared according to the general procedure D, using a solution of 2 g (1 equiv, 1 mmol, 0.28231 g). Yield = 79% (was obtained as a white solid); R*_f_* = 0.20 [SiO_2_, CH_2_Cl_2_]; Mp 91–92°C. IR (KBr, *ν* cm^−1^): 1382 and 1134 (SO_2_), 1729 and 1748 2(C=O). ^1^H NMR (300 MHz, CDCl_3_): 1.28 (t, 3H, CH_3_), 1.41 (s, 9H, *t*Bu), 3.77 (s, 2H, CH_2_CO), 4.23 (q, 2H, CH_2_-O), 4.72 (s, 2H, CH_2_-N), 4.88 (s, 2H, CH_2_-N-Boc), 7.20–7.35 (m, 4H, H-Ar). HRMS ESI^+^: *m/z*: 407 [M+Na]^+^, 791[M*2+Na]^+^.

**1,5-dihydro-2-tert-butoxycarbonyl-4-(benzyl(methoxycarbonyl)methyl)benzo[d][1,2,7]thiadiazepine-3,3-dioxides (3d)**

This compound was prepared according to the general procedure D, using a solution of 2 h (1 equiv, 1 mmol, 0.35841 g). Yield = 76% (was obtained as a viscous oil); R*_f_* = 0.37 [SiO_2_, CH_2_Cl_2_]. IR (KBr, *ν* cm^−1^): 1368 and 1142 (SO_2_), 1735 and 1742 (C=O). ^1^H NMR (300 MHz, CDCl_3_): 1.40 (s, 9H, *t*Bu), 3.25 (s, 3H, CH_3_–O), 5.01 (t, 1H, CH), 4.76 (d, 2H, CH_2_-Ph), 4.73 (s, 2H, CH_2_–N–Boc), 4.65 (s, 2H, CH_2_–N), 7.08–7.28 (m, 9H, Ar-H). HRMS ESI^+^: *m/z*: 483 [M+Na]^+^, 944[M*2+Na]^+^.

**1,5-dihydro-2-tert-butoxycarbonyl-4-(phenyl(methoxycarbonyl)methyl)benzo[d][1,2,7]thiadiazepine-3,3-dioxides (3e)**

This compound was prepared according to the general procedure D, using a solution of** 2i **(1 equiv, 1 mmol, 0.34438 g). Yield = 66% (was obtained as a white solid); R*_f_* = 0.38 [SiO_2_, CH_2_Cl_2_]; Mp: 90–92°C. IR (KBr, *ν* cm^−1^): 1314 and 1138 (SO_2_), 1732 and 1708 (C=O). ^1^H NMR (300 MHz, CDCl_3_): 1.43 (s, 9H, *t*Bu), 3.66 (s, 3H, CH_3_-O), 5.96 (s, 1H, CH), 6.98–7.32 (m, 9H, H-Ar), 4.38–4.80 (dd, 2H, CH_a_H_b_-N), 4.85–5.05 (dd, 2H, CH_a_H_b_-N-Boc), 6.98–7.32 (m, 9 h, H-Ar). HRMS ESI^+^: *m/z*: 469 [M+Na]^+^, 915[M*2+Na]^+^.

### 5.6. General Procedure E for the Synthesis of Fused Benzothiadiazepines **(4a-f)**

An acetonitrile solution (50 mL) containing *N*,*N*′-disubstituted symmetric sulfamide (2 equiv, 2 mmol), 2,3,4,5-tetrakis(bromomethyl)benzene (1 equiv, 1 mmol, 0.44980 g), and anhydrous K_2_CO_3_ (4.5 equiv, 4.5 mmol, 0.6219 g) was heated at reflux for 10 hours. The reaction was followed by TLC (CH_2_Cl_2_). On completion, the reaction mixture was cooled to room temperature, filtered, and after removal of the solvent under reduced pressure a solid was obtained. The solid was redissolved in dichloromethane (CH_2_Cl_2_), washed with 2 portions of HCl 1 M (2 × 20 mL) followed with water (2 × 30 mL), and dried with Na_2_SO_4_. The solution was filtered and concentrated under reduced pressure to leave a crude product. The residue was purified by chromatography on silica gel using CH_2_Cl_2_ to yield fused symmetric and asymmetric benzothiadiazepines

**Compound (4a)**

This compound was prepared according to the general procedure E, using a solution of **1a** (2 equiv, 2 mmol and 0.36054 g). Yield = 76% (was obtained as a white solid); R*_f_* = 0.25 [SiO_2_, CH_2_Cl_2_]; Mp 264–266°C. IR (KBr, *ν* cm^−1^): 1339 and 1148 (SO_2_). ^1^H NMR (300 MHz, CDCl_3_): 0.90 (t, 12H, CH_3_), 1.58 (m, 8H, *β*-CH_2_), 2.91 (t, 8H, *α*-CH_2_), 4.46 (s, 8H, N-CH_2_-Ar), 7.15 (s, 2H, H-Ar). ^13^C NMR (CDCl_3_): 11.02 (*γ*-C), 21.08 (*β*-C), 48.90 (*α*-C), 50.03 (CH_2_), 132.50 and 136.85 (CAr). LRMS (CI): 487 [M+H]^+^; HRMS ESI^+^: m/z: 509 [M+Na]^+^.

**Compound (4b)**

This compound was prepared according to the general procedure E, using a solution of** 1b **(2 equiv, 2 mmol and 0.41664 g). Yield = 79% (was obtained as a white solid); R*_f_* = 0.29 [SiO_2_, CH_2_Cl_2_]; Mp 141–143°C. IR (KBr, *ν* cm^−1^): 1354 and 1149 (SO_2_). ^1^H NMR (300 MHz, CDCl_3_): 0.91 (t, 12H, CH_3_), 1.34 (m, 8H, *γ*-CH_2_), 1.52 (m, 8H, *β*-CH_2_), 2.94 (t, 8H, *α*-CH_2_), 4.46 (s, 8H, N-CH_2_), 7.16 (s, 2H, H-Ar). ^13^C NMR (CDCl_3_): 13.68 (*δ*-C), 19.58 (*γ*-C), 29.79 (*β*-C), 46.82 (*α*-C), 49.97 (CH_2_), 132.49 and136.85 (CAr). LRMS (CI): 543 [M+H]^+^; HRMS ESI^+^: m/z: 567 [M+Na]^+^.

**Compound (4c)**

This compound was prepared according to the general procedure E, using a solution of **1d** (2 equiv, 2 mmol and 0.5527 g). Yield = 75% (was obtained as a white solid); R*_f_* = 0.46 [SiO_2_, CH_2_Cl_2_]; Mp 313–314°C. IR (KBr, *ν* cm^−1^): 1364 and 1157 (SO_2_). ^1^H NMR (300 MHz, CDCl_3_): 4.25 (s, 8H, CH_2_-Ph), 4.35 (s, 8H, N-CH_2_), 6.79 (s, 2H, H-Ar), 7.28–7.37 (m, 20H, Ph). HRMS ESI^+^: *m/z*: 701 [M+Na]^+^.

**Compound (4d)**

This compound was prepared according to the general procedure E, using a solution of **2a** (2 equiv, 2 mmol, 0.5727 g). Yield = 55% (was obtained as a white solid); R*_f_* = 0.22 [SiO_2_, CH_2_Cl_2_]; Mp 160–162°C. IR (KBr, *ν* cm^−1^): 1392 and 1150 (SO_2_), 1715 (C=O). ^1^H NMR (300 MHz, CDCl_3_): 1.42 (s, 18H, *t*Bu), 4.05 (s, 4H, CH_2_-N-Bn), 4.39 (s, 4H, CH_2_-N-Boc), 4.90 (s, 4H, CH_2_-Ph), 7.13 (s, 2H, H-Ar), 7.36–7.44 (m, 10H, Ph). HRMS ESI^+^: *m/z*: 721 [M+Na]^+^.

**Compound (4d′)**

Yield = 15% (was obtained as a white solid); R*_f_* = 0.25 [SiO_2_, CH_2_Cl_2_]; Mp 159–161°C. IR (KBr, *ν* cm^−1^): 1371 and 1155 (SO_2_), 1715 (C=O). ^1^H NMR (300 MHz, CDCl_3_): 1.45 (s, 18H, *t*Bu), 4.17 (s, 4H, CH_2_-N-Bn), 4.40 (s, 4H, CH_2_-N-Boc), 4.90 (s, 4H, CH_2_-Ph), 6.75 (s,1H, H-Ar), 7.27 (s, 1H, H-Ar), 7.36–7.44 (m, 10H, Ph). HRMS ESI^+^: *m/z*: 721 [M+Na]^+^.

**Compound (4f)**

This compound was prepared according to the general procedure E, using a solution of **2j **(2 equiv, 2 mmol, 0.5646 g). Yield = 37% (was obtained as a white solid); R*_f_* = 0.20 [SiO_2_, CH_2_Cl_2_]; Mp 169–171°C. IR (KBr, *ν* cm^−1^): 1368 and 1144 (SO_2_), 1732 and 1745 (C=O). ^1^H NMR (300 MHz, CDCl_3_): 1.44 (s, 18H, *t*Bu), 1.34 (d, 6H, CH_3_), 3.53 (s, 6H, O-CH_3_), 4.65 (q, 2H, CH), 4.86 (s, 4H, N-CH_2_-Ar), 5.00 (s, 4H, CH_2_-N-Boc), 7.13 (s, 2H, H-Ar). HRMS ESI^+^: *m/z*: 713 [M+Na]^+^.

**Compound (4f′)**

Yield = 34% (was obtained as a white solid); R*_f_* = 0.24 [SiO_2_, CH_2_Cl_2_]; Mp 168–170°C. IR (KBr, *ν* cm^−1^): 1390 and 1144 (SO_2_), 1732 and 1750 (C=O). ^1^H NMR (300 MHz, CDCl_3_): 1.42 (s, 18H, *t*Bu), 1.30 (d, 6H, CH_3_), 3.47 (s, 6H, O-CH_3_), 4.63 (q, 2H, CH), 4.84 (s, 4H, N-CH_2_-Ar), 4.96 (s, 4H, CH_2_-N-Boc), 7.00 (s, 1H, H-Ar), 7.24 (s, 1H, H-Ar). HRMS ESI^+^: *m/z*: 713 [M+Na]^+^.

**Compound (4e)**

This compound was prepared according to the general procedure E, using a solution of 2 g (2 equiv, 2 mmol, 0.5646 g). Yield = 48% (was obtained as a white solid); R*_f_* = 0.18 [SiO_2_, CH_2_Cl_2_]; Mp 181–183°C. IR (KBr, *ν* cm^−1^): 1380 and 1139 (SO_2_), 1723 and 1751 (C=O). ^1^H NMR (300 MHz, CDCl_3_): 1.40 (s, 18H, *t*Bu), 1.29 (t, 6H, CH_3_), 3.75 (s, 4H, CH_2_CO), 4.22 (q, 4H, CH_2_CH_3_), 4.73 (s, 4H, CH_2_), 4.86 (s, 4H, CH_2_-N-Boc), 7.21 (s, 2H, H-Ar). HRMS ESI^+^: *m/z*: 713 [M+Na]^+^.

**Compound (4e′)**

Yield = 24% (was obtained as a white solid); R*_f_* = 0.22 [SiO_2_, CH_2_Cl_2_]; Mp 179–181°C. IR (KBr, *ν* cm^−1^): 1380 and 1139 (SO_2_), 1723 and 1755 (C=O). ^1^H NMR (300 MHz, CDCl_3_): 1.43 (s, 18H: *t*Bu), 1.28 (t, 6H: CH_3_), 3.77 (s, 4H, CH_2_CO), 4.20 (q, 4H, CH_2_CH_3_), 4.71 (s, 4H, CH_2_), 4.90 (s, 4H, CH_2_-N-Boc), 7.05 (s, 1H, H-Ar), 7.32 (s, 1H, H-Ar). HRMS ESI^+^: *m/z*: 713 [M+Na]^+^.

### 5.7. General Procedure F for the Synthesis of New Macrocyclic Sulfamide **5a**

A solution of 1-methoxy-4-*tert*-butyl-(2,6-dibromomethyl)benzene (1 equiv, 1 mmol, 0.350 g) in 20 mL of anhydrous CH_3_CN and a solution of *N*,*N*-disubstituted sulfamide **1d** (1 equiv, 1 mmol, 0.2763 g) in CH_3_CN (20 mL) were slowly added by syringe pump over several 5 hours at the same rate to a mixture of K_2_CO_3_ (4.5 equiv, 4.5 mmol, 0.622 g) and CH_3_CN (150 mL). After stirring at reflux for 24 h, the reaction mixture was filtered and the filtrate was concentrated *in vacuum*. The residue was dissolved in CH_2_Cl_2_, washed with HCl (0.1 N) (2 × 20 mL) water (2 × 30 mL), and dried with Na_2_SO_4_ and the solvent was evaporated under reduced pressure. The residue was purified by chromatography on silica gel using CH_2_Cl_2_ to yield the pure macrocyclic sulfamide **5a**.

**Macrocyclic Sulfamide (5a)**

Yield = 58% (was obtained as a white solid); R*_f_* = 0.47 [SiO_2_, CH_2_Cl_2_]; Mp > 350°C. IR (KBr, *ν* cm^−1^): 1361 and 1148 (SO_2_). ^1^H NMR (300 MHz, CDCl_3_): 1.35 (s, 18H, *t*Bu), 3.10 (s, 6H, CH_3_), 3.8 (br, 8H, N-CH_2_), 4.85 (br, 8H, CH_2_-Ph), 6.78 (s, 4H, H-Ar), 7.30–7.45 (m, 20H, Ph). HRMS ESI^+^: *m/z*: 952 [M+Na]^+^.

## Figures and Tables

**Table 1:** IR, ^1^H NMR, and mass spectral data for compounds **4a-f **and **4d′-f′**.

C*omp. *	R	RMN^1^H signals for aromatic region (*δ* : ppm)		Yield [%]	ESI (m/z)	IR (KBr, *ν* cm^−1^)	
						**SO_2_**	**C=O**
**4a**	*Propyl*	7.15 (s, 2H)		76	509	1148, 1339	/
**4b**	*Butyl*	7.16 (s, 2H)		79	567	1149, 1354	/
**4c**	*Benzyl*	6.79 (s, 2H)		75	710	1157, 1357	/
**4d**	*Benzyl*	7.13 (s, 2H)		55	721	1150, 1392	1715
**4e**	*CH_2_COOE*t	7.21 (s, 2H)		48	713	1139, 1380	1723, 1751
4**f**	*CH(CH_3_)COOMe*	7.13 (s, 2H)		37	713	1144, 1368	1732, 1745
4**d**′	*Benzyl*	6.75 (s, 1H)	7.27(s, 1H)	15	721	1155, 1371	1715
4**e**′	*CH_2_COOE*t	7.05 (s, 1H)	7.32 (s, 1H)	24	713	1139, 1380	1723, 1755
4**f**′	*CH(CH_3_)COOMe*	7.00 (s, 1H)	7.24(s, 1H)	34	713	1144, 1390	1732, 1750
